# Identification of a seven-gene prognostic model for renal cell carcinoma associated with CD8+T lymphocyte cell

**DOI:** 10.1097/MD.0000000000039938

**Published:** 2024-10-04

**Authors:** Jingbang Liu, Tao Jiang

**Affiliations:** a Department of Urology, The Second Affiliated Hospital of Dalian Medical University, Dalian, China.

**Keywords:** bioinformatics, CD8 + T lymphocytes, renal cell carcinoma, tumor adaptive immunity, tumor microenvironment

## Abstract

CD8+ T lymphocytes are important elements of the tumor microenvironment, hence their involvement in the development and progression of tumors is complex. Data on the precise tumor-infiltrating lymphocytes gene signature in renal cell carcinoma (RCC) remain limited. Therefore, this study created a tumor-infiltrating lymphocytes-related predictive model for patients with RCC using data from The Cancer Genome Atlas. The most important genes associated with CD8 + T lymphocytes were identified using weighted gene co-expression network analysis. Functional categories of important genes were revealed using gene ontology enrichment and Kyoto Encyclopedia of Genes and Genomes signaling pathway analyses. A CD8 + T lymphocyte-related prognostic model with 7 important genes was simultaneously created using the least absolute shrinkage and selection operator, univariate and multivariate Cox regressions, and the 7 genes were expressed particularly in CD8 + T lymphocytes according to single-cell sequencing data obtained from the Gene Expression Omnibus. This study identified a seven-gene prognostic model associated with CD8 + T lymphocytes that may significantly influence risk stratification in patients with RCC. The genes included in the model are apolipoprotein B mRNA editing catalytic polypeptide 3G, CD3 gamma, eomesodermin, protein tyrosine phosphatase, non-receptor type 7, signal regulatory protein gamma, Fas ligand, and T-cell immunoreceptor with Ig and ITIM domains.

## 1. Introduction

An estimated 14,890 individuals will die from renal cell carcinoma (RCC) and 81,800 new cases of RCC are anticipated in the United States by the end of 2023.^[1]^ Although most epidemiological data are general for RCC, its histologically accounts for the vast majority (90%) of reported cases.^[2]^ RCC originates from the renal tubular epithelial cells that line the proximal convoluted tubules and tiny kidney conduits that carry urine.^[3]^ High blood pressure, obesity, and smoking increase the risk of RCC.^[4]^ Approximately 50% of RCC cases are accidentally discovered upon medical checkups, with approximately 25% of patients diagnosed with metastatic disease.^[5]^ The 5-year relative survival rates through surgical treatment and adjunctive therapy have shown some improvement; however, the overall prognosis remains poor, particularly for metastatic RCC.^[6]^ Traditional treatment methods, such as surgery and chemotherapy, have shown unsatisfactory results.^[6]^ In 2012, the International Society of Urological Pathology implemented a novel classification system for RCC, principally centered around the evaluation of nucleoli. This approach categorizes RCC into 4 distinct classes.^[7]^

The tumor microenvironment (TME) of RCC is characterized by the presence of tumor-infiltrating lymphocytes (TIL), particularly CD8 + T lymphocytes, which play a crucial role in the immune response against tumor-associated antigens (TAA). The microenvironment of cancer, regulating its progression and showing TIL form an ecosystem in the potential prognostic value.^[8]^ TME of RCC consists primarily of T cells, natural killer (NK) cells, B cells, macrophages, and dendritic cells, all of which are involved in complex interactions.

For example, tumor-associated macrophages suppress T-cell activity by secreting immunosuppressive factors such as IL-10 and TGF-β, thereby reducing the body’s immune response to tumors. Tumor-associated macrophages can also support tumor growth by promoting angiogenesis, for instance, through the secretion of vascular endothelial growth factor. Additionally, they may accelerate tumor progression by facilitating the migration and invasiveness of tumor cells.^[9]^

Cytotoxic CD8 + T lymphocytes and CD4 + helper T cells can attack antigenic tumor cells and prevent the spread of cancer,^[10]^ while antigenic tumor cells can be targeted by these cells to prevent tumor growth. A strong correlation has been reported between the immune response and clinical outcome of RCC.

Due to the heterogeneity of RCC, useful biomarkers that contribute to individualized treatment remain lacking, particularly in immunotherapy.^[8]^ As a characteristic of TME, RCC cells are prone to immune infiltration. CD8 + T lymphocytes aid in tumor adaptive immunity and constitute the greatest fraction of RCC immune cells;^[8]^ however, poor prognosis is linked to increased CD8 + T lymphocyte infiltration in RCC.^[11]^ Mutations in the von Hippel–Lindau gene lead to the accumulation of hypoxia-inducible factor α (HIFα) within cells, which in turn stimulates the production of factors such as vascular endothelial growth factor, promoting cancer progression.^[12]^Seven medications that target the von Hippel–Lindau signaling pathway have been approved by the Food and Drug Administration, although data that support their use in improving clinical outcomes and survival in patients with RCC are limited.^[13]^

Therefore, further research on the development and prognosis prediction of RCC is warranted to gain deeper insights into its pathogenesis at the molecular level and identify new targets for therapeutic intervention.

In recent years, bioinformatics technology has facilitated the development of numerous tools to identify biomarkers. In this study, we emphasize that TILs localized to TAA are pivotal in modulating the immune landscape of RCC. Our analysis reveals that CD8 + T lymphocytes constitute a significant proportion of the immune cell population within the TME, highlighting their potential as therapeutic targets.

we identified CD8 + T lymphocyte-related biomarkers of RCC using bioinformatics technology, including apolipoprotein B mRNA editing catalytic polypeptide 3G (*APOBEC3G*), CD3 gamma (*CD3G*), eomesodermin (*EOMES*), protein tyrosine phosphatase, non-receptor type 7 (*PTPN7*), signal regulatory protein gamma (*SIRPγ*), Fas ligand (*FASLG*) and T-cell immunoreceptor with Ig and ITIM domains (*TIGIT*).

## 2. Methods

### 2.1. Data collection

We have completed the STROBE Statement. FPKM-uq gene expression data (607 samples), survival data (979 samples), and phenotypic data (985 samples) of patients diagnosed with RCC were downloaded from The Cancer Genome Atlas (TCGA) database (https://xena.ucsc.edu), which is a landmark cancer genomics project. To develop a gene signature and investigate prognostic survival, we retrieved clinical data, including ID, age, stage, T, N, histologic grade, sex and overall survival (OS). T represents the primary tumor, N indicates lymph node involvement, and M denotes metastatic spread.

Samples with variables having missing values were excluded. GSM4630028 (RCC sample) and GSM4630031 (normal kidney tissue) was downloaded from the Gene Expression Omnibus (https://www.ncbi.nlm.nih.gov/geo/) database for single-cell RNA sequence analysis.

### 2.2. Statistical analysis

R software (version 4.3.1) and R studio were used for statistical analyses and generating images. Microsoft Excel was also used for the statistical analysis. *P* < .05 is considered statistically significant.

### 2.3. Immune infiltration analysis by CIBERSORT

We annotate the ENSEMBL-ID of RCC downloaded from TCGA with R software packages “mapids.” The results of CIBERSORT were then calculated using the R software package “cibersort.” The TIL proportion was then displayed using the R software package “ggplot2.”

### 2.4. Weighted correlation network analysis

Correlation networks have become popular in bioinformatics applications. Weighted gene co-expression network analysis (WGCNA) is a systems biology tool for describing gene correlation patterns across microarray samples.^[14]^ We included the genes with the top 10,000 mad values into the calculation using the R package “WGCNA,” clustered the samples and eliminated obvious outlier samples. Pearson correlation values were obtained to construct a gene matrix. Next, we performed a network topology analysis to determine the appropriate soft threshold for building a scale-free network with good connectivity and subsequently used a one-step method to construct the network and perform module detection. The results obtained by CIBERSORT, deemed as phenotypic data, were correlated with the clustering module generated using WGCNA. We selected the module with the highest phenotypic correlation and identified the relationship between gene significance (GS) and module membership (MM) in the module. According to the GS and MM results, values were set to identify hub genes, and a visual feature gene network was created.

### 2.5. Identification of hub genes and enrichment analysis

MM > 0.85 and gene GS > 0.65 were set as candidate hub genes. Following hub genes identification, the String Database (https://cn.string-db.org/) was used to perform PPI network analysis to investigate their interactions. The interaction with TIL-related genes was shown using the Cytoscape software. Gene ontology (GO) and Kyoto Encyclopedia of Genes and Genomes enrichment analyses were processed by R software packages “clusterProfiler”

### 2.6. Construction and validation of gene prognostic model

We combined TIL-related gene expression data with survival data. According to R “sample” function, the 602 patients were randomly separated into training and validation cohorts at a 1:1 ratio. Least absolute shrinkage and selection operator (LASSO)-Cox regression was used to select the genes. Model overfitting was avoided by using the LASSO-Cox model, which can also be used to adjust the model to avoid overcompression of coefficients. Seven genes were selected, the coefficients of which were extracted to determine the risk score for each patient. The calculation for the risk score was as follows: risk score = ∑Expression (mRNAi) × Coefficient (mRNAi), which was tested in the validation cohorts. Using the median risk score from the training cohort as the basis, we classified the validation cohort into high- and low-risk categories and divided the test cohort into 2 groups according to the median risk score of the training cohort; the ROC and Kaplan–Meier survival curves were analyzed to assess the model’s efficacy. Finally, we used univariate and multivariate Cox regression analyses with the risk score as an independent variable. After univariate Cox analysis, variables with *P* > .05. Nomograms provide a clear explanation of the prognostic model. To create a nomogram that incorporated risk score, age, and stage, we used the regplot function in the R software. We plotted ROC curves of the nomogram in the training cohort to evaluate the model.

### 2.7. Using external dataset for validation

To prevent false positive results in the calculation process, we used the GEPIA database (http://gepia.cancer-pku.cn) to verify and draw a box diagram of gene expression and identified common transcription factors of hub gene on the ChEA3 database (https://maayanlab.cloud). By comparing several pathological kinds of RCC with healthy kidneys, Cheng Su et al have clearly shown tumor features at the scRNA-seq.^[15]^ Hence, we obtained 2 samples from GSE152938: a RCC sample (GSM4630028) and normal kidney tissue (GSM4630031) and used the Seurat R package to perform cell annotation to control data quality and reduce data dimensions. Using the Seurat package, we also validated the expression of hub genes in the TIL of RCC.

## 3. Results

### 3.1. Tumor immune cell infiltration in RCC

**Figure 1.**
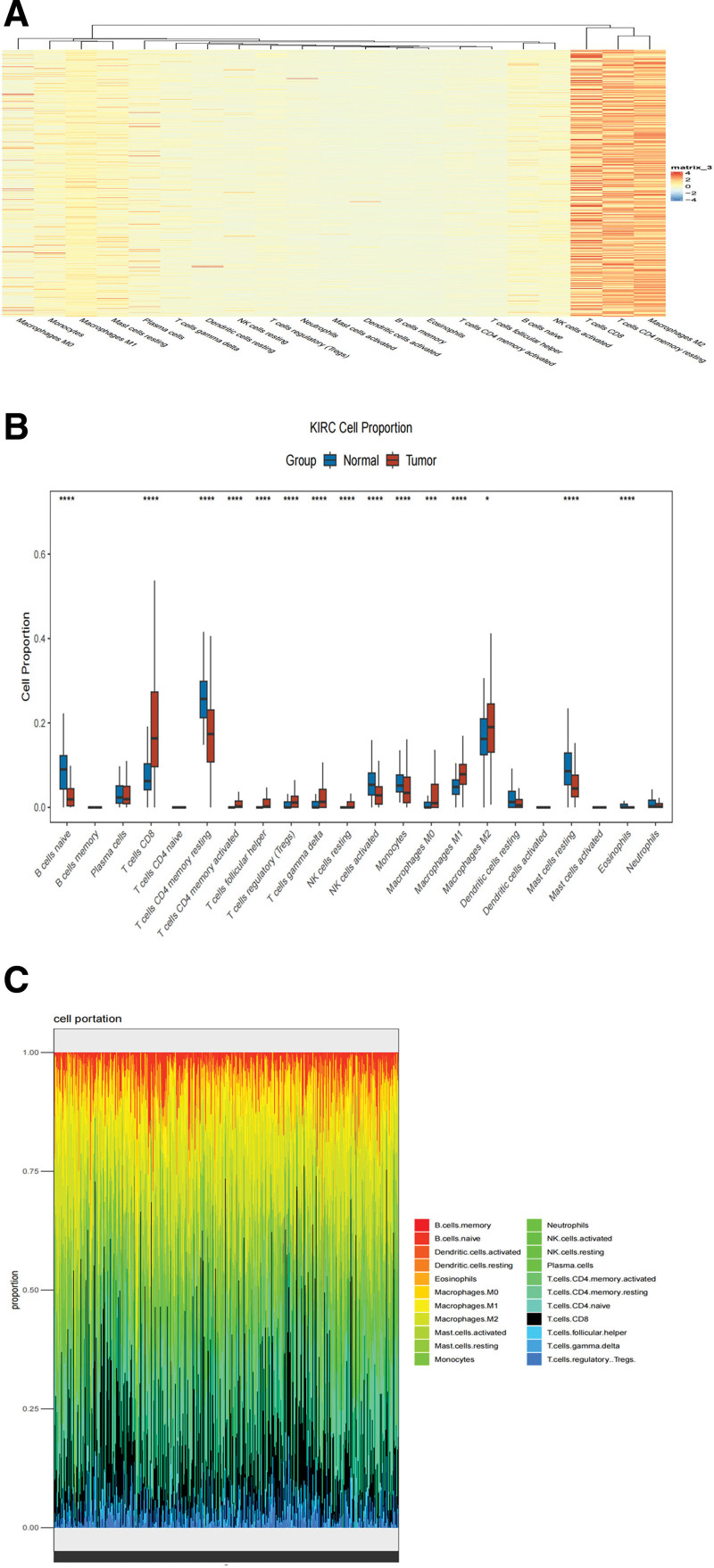
TIL in TME based on TCGA-RCC cohort (607 patients). (A) The proportion of each immune cell following standardization. (B) A column chart showing TIL in normal and tumor groups. (C) A stacking ratio diagram showing TIL in patients with RCC.

The percentage of immune cell infiltration in each sample was normalized. Figure 1A illustrates the high percentage of CD8 + T-cells in the sample. Next, we divided the samples into an experimental group (tumor) and a control group (normal) and calculated the proportion of each immune cell in the RCC immune microenvironment (Fig. 1B). We also calculated the accumulation ratio of tumor immune cells. CD8 + T lymphocytes are indicated in black in Figure 1C. CD8 + T lymphocytes accounted for the highest proportion of some tumor samples, even more than the sum of the proportions of all other immune cells. We also calculated the proportion of each type of immune cell present in the entire tumor microenvironment: T cells CD8: 0.192729, T cells CD4 naive: 0, T cells CD4 memory resting: 0.186887, T cells CD4 memory activated: 0.010546, macrophages M1: 0.076427, macrophages M2: 0.194426, dendritic cells resting: 0.015531, dendritic cells activated: 0.003556. The metastasis status of 31 patients was unclear, and data for 2 patients were missing and 97 patients had distant tumor metastasis.

### 3.2. Identification of TIC-related gene modules using WGCNA

A total of 607 samples from TCGA-RCC sequencing were available for WGCNA to identify cell-specific gene sets efficiently and accurately. Five outlier samples were eliminated, and the samples were linked to the CIBERSORT results. We visualized the relationship between the TIL and the sample tree (Figure S1, Supplemental Digital Content, http://links.lww.com/MD/N680).

**Figure 2.**
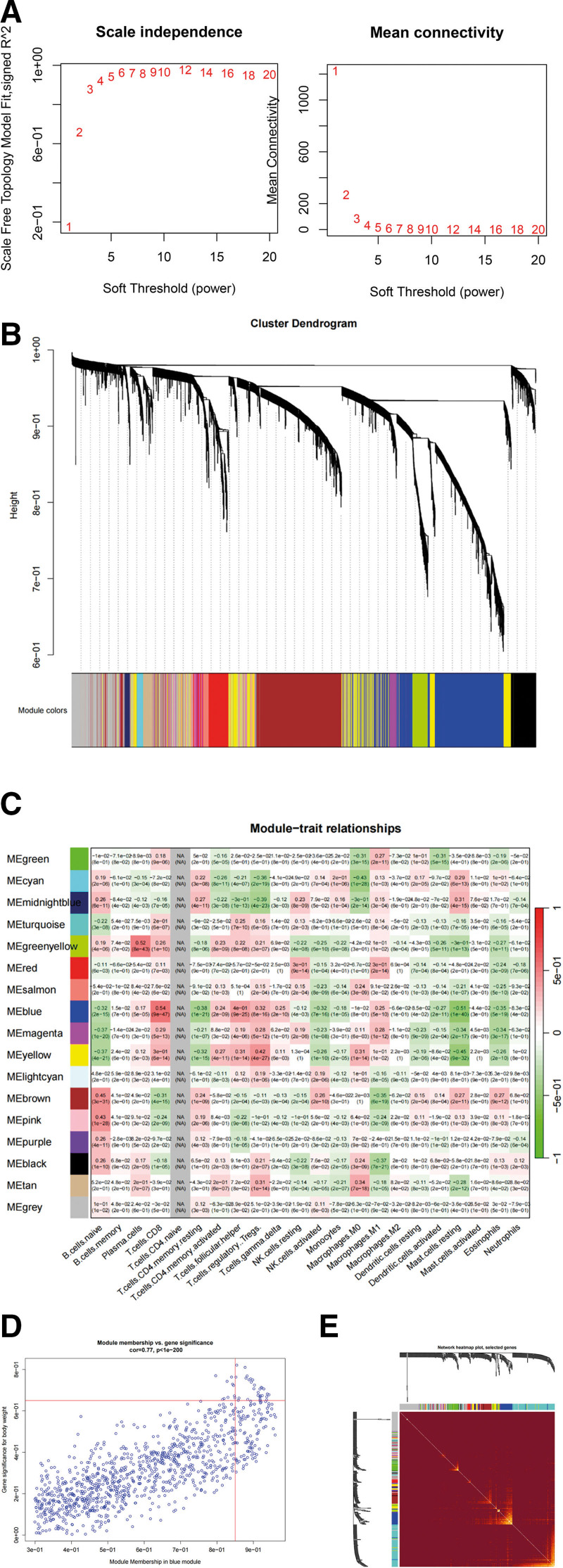
Weighted gene co-expression network analysis. (A) Soft thresholds and scale-free network validation. The soft threshold is selected as 3. (B) Cluster dendrogram with the gene modules. (C) Correlation between gene modules and immune cell fraction. (D) Correlation between GS and MM. (F) Network heatmap of selected genes.

The scales were determined to be independent of each other. A topological overlap matrix was created to gauge network connectedness (Fig. 2A). We selected the soft threshold power β = 4 to calculate the weight parameters of the adjacency matrix for the subsequent construction of a weighted gene network. The hierarchical clustering tree diagram of module recognition is shown in Figure 2B. The proportion of colorless gene modules was <20% of the total. The number of modules and features was moderate. Each association was color-coded using correlation values (Fig. 2C). Naïve CD4 + T cells were not present in the tumor microenvironment of RCC, hence no color was observed. The highest correlation coefficient was observed for the blue module, which had the closest relationship with CD8 + T lymphocytes. The gene in the blue module was extracted. Under the conditions of GS > 0.65 and MM > 0.85, we identified the hub genes in the blue gene module (Fig. 2D). One method to visualize a weighted network is to draw a heatmap in which each row and column corresponds to a gene. Heatmaps describe adjacent or topological overlaps. Light colors represent low adjacency (overlap), whereas dark represent high adjacency (overlap). The gene tree and module colors were drawn along the top and left sides of the heatmap, and the blue module had a low degree of overlap (Fig. 2E).

### 3.3. Gene enrichment and construction of the seven-gene prognostic model

**Figure 3.**
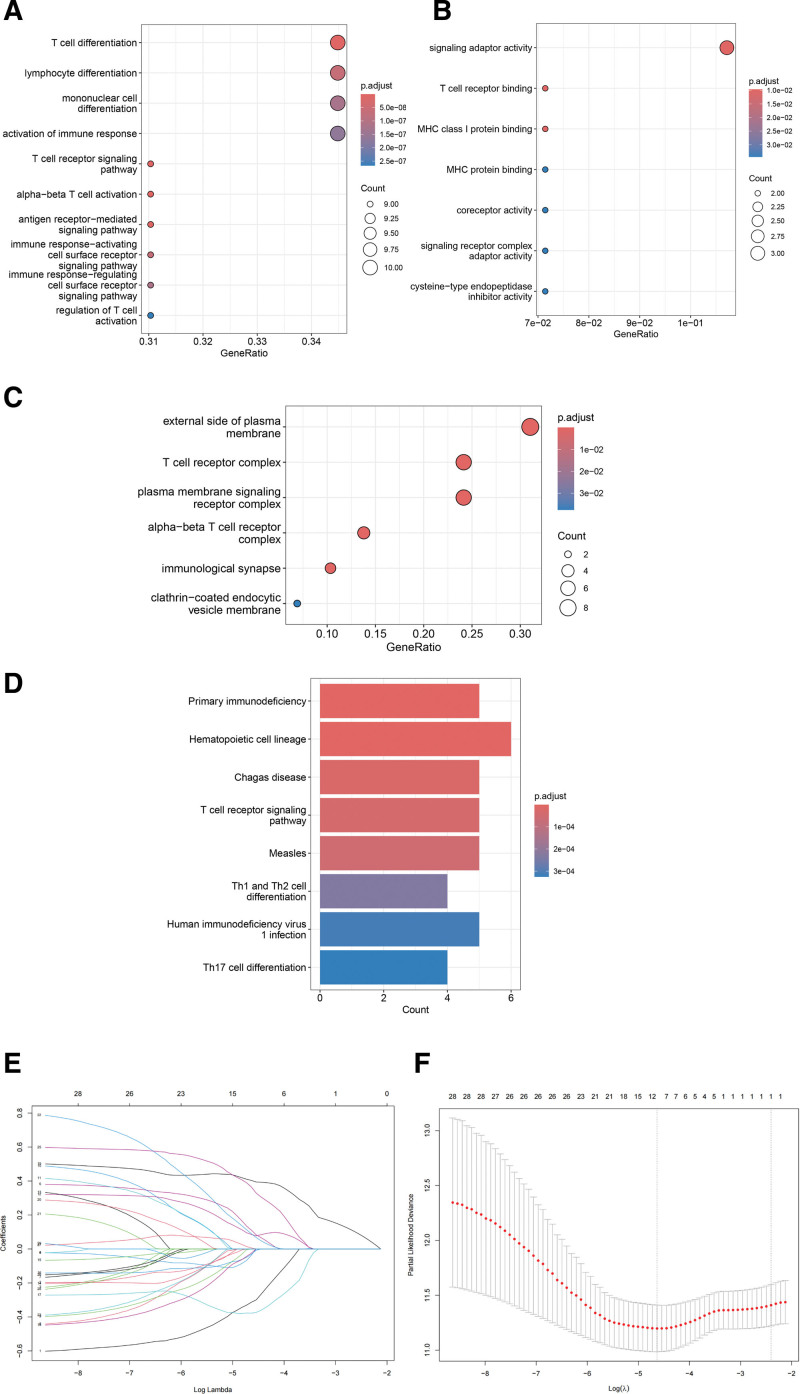
Gene enrichment and LASSO analysis. (A) Biological process of gene ontology enrichment (GO). (B) Cellular component of GO. (C) Molecular function of GO. (D) Kyoto Encyclopedia of Genes and Genomes (KEGG) signaling pathway analysis for 19 hub genes. (E) LASSO regression analysis was used to identify the 7 genes signature. (F) Cross-validation in the LASSO model.

Using the String Database (http://string-db.org/), we examined the protein-protein interaction (PPI) networks of 19 genes. The aforementioned hub genes were displayed using the Cytoscape program (Figure S2, Supplemental Digital Content, http://links.lww.com/MD/N681). Additionally, we carried out GO enrichment analysis for the 19 hub genes, which included biological process (Fig. 3A), cellular component (Fig. 3B), and molecular functions (Fig. 3C), as well as Kyoto Encyclopedia of Genes and Genomes signaling pathway analysis (Fig. 3D). The genes in the blue model were correlated with T-cell differentiation, lymphocyte differentiation, external side of the plasma membrane, signaling adaptor activity, primary immunodeficiency, and hematopoiesis and were filtered using Lasso-Cox regression (Fig. 3E and F).

When the lambda had the minimum value, we extracted the genes used to construct the model; thus, only 7 genes were selected from the analysis, namely *EOMES, SIRPG, PTPN7, CD3G, APOBEC3G, FASLG* and *TIGIT*, the coefficients of which were −0.23379373, 0.04964700, 0.09033733, −0.34412765, 0.39095263, 0.03301778, and 0.287, respectively. Furthermore, the following formula was used to determine the risk score for 602 patients:


$$\begin{array}{l} \begin{array}{l} Risk\;score=EOMES*-0.23379373\;+SIRPG*0.04964700\\ +PTPN7*0.09033733\;+CD3G*-0.34412765\\ +APOBEC3G*0.39095263+FASLG*0.03301778+TIGIT*0.287\; \end{array} \end{array}$$

### 3.4. Evaluation of prognostic models and nomogram

**Figure 4.**
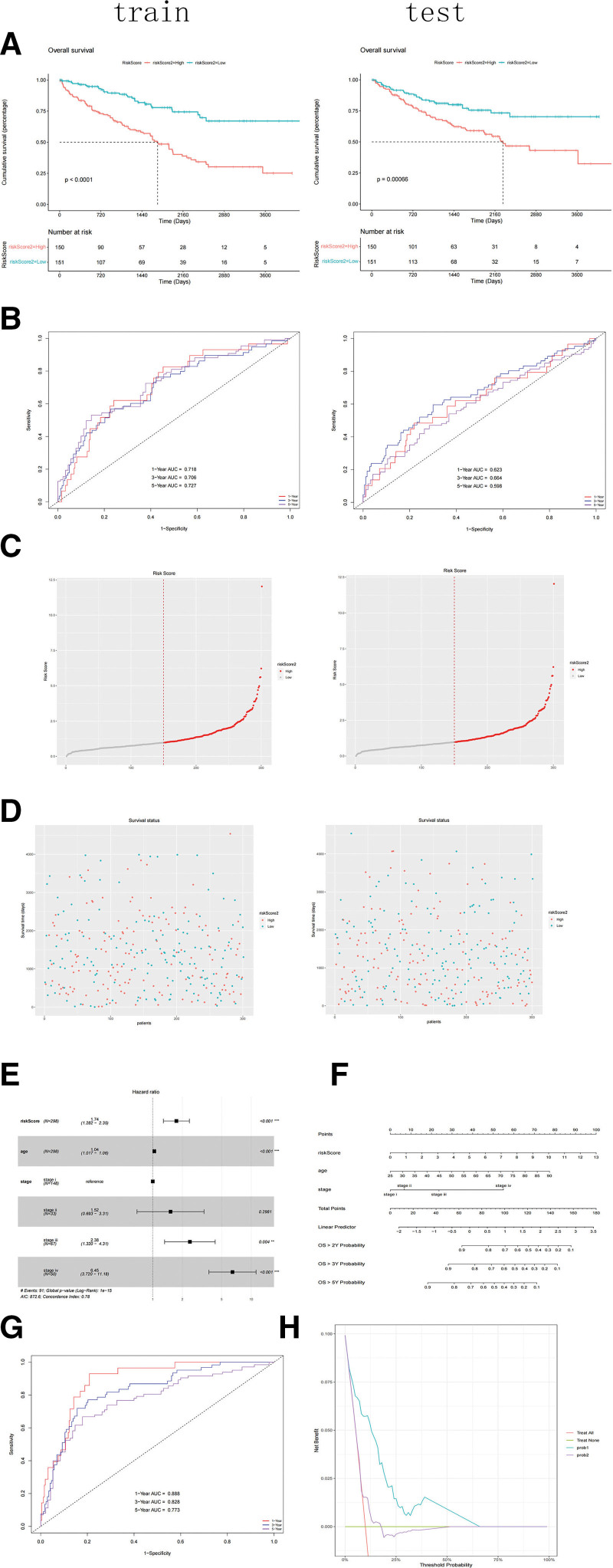
Evaluation validation of the 7 gene signature. (A) Kaplan–Meier survival curve in train and test cohorts. (B) TME-dependent ROC curve of our gene signature in train and test cohorts. (C) Risk score curve in train and test cohorts. (D) Patients’ status as risk score increases in train and test cohorts. (E) Multivariate Cox analysis in the train and test cohorts. (F) Nomogram of our multivariate cox analysis. (G) TME-dependent ROC curve of our multivariate cox analysis in the training and test cohorts.. (H) DCA curve of multivariate cox analysis (prob1) and our gene signature (prob2) in training cohort.

The high- and low-risk patient groups were separated into training cohorts. Differences between the 2 groups were examined using the Kaplan–Meier method with the log-rank test (Fig. 4A). Patients in the low-risk group lived longer than those in the high-risk. To further confirm the accuracy of our model, a TME-dependent ROC curve (Fig. 4B) was created. Nearly 0.72 square feet covered the train’s ROC curve. Figure 4C and D shows the risk curve and survival status plot, respectively. The results indicated that the patient’s risk and risk score were positively correlated. We performed univariate analyses using the risk score as an independent variable in combination with other clinical variables and concluded that the risk score based on the 7 genes, age, and stage was an independent predictive indicator (*P* < .05). We used these 3 parameters for the multivariate Cox analysis and constructed forest plots (Fig. 4E). Finally, a nomogram (Fig. 4F) was developed to display the multivariate Cox regression results and provide a reference model for forecasting the survival rates of patients with RCC at 1, 3, and 5 years. We evaluated the ROC curve of the nomogram in the training and test cohorts (Fig. 4G) and generated a DCA curve (Fig. 4H) to compare the performance of the nomogram and risk-scoring models. Nearly 0.9 square feet covered the train’s ROC curve and the test’ ROC curve; prob1 represents the nomogram, while prob2 represents the risk score model.

### 3.5. GEPIA database and RCC single-cell RNA-seq-for validation

**Figure 5.**
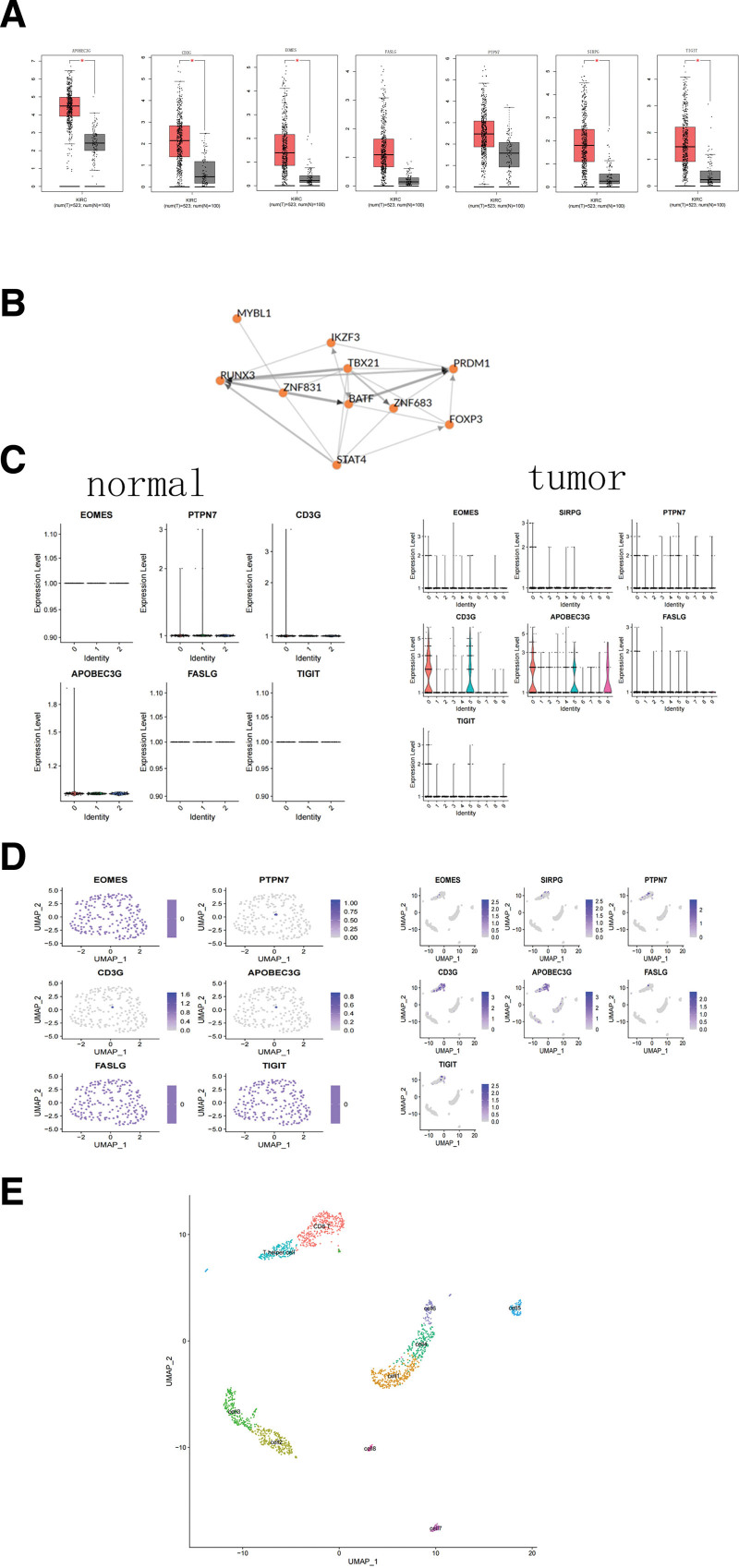
(A) Expression of the 7 genes of different tissues in the GEPIA database. (B) Common transcription factors of the 7 genes based on the ChEA3 database. (C) Expression level of the 7 genes based on scRNA-seq. (D) Umap plots of the 7 genes based on scRNA-seq in GSM4630028. (E) Heatmap of expression level of the 7 genes.

The GEPIA database has been previously developed by Zemin et al at Peking University in 2017 and includes RNA sequencing data of 9736 tumor tissues and 8587 normal tissues from TCGA and GTEx databases. We input the 7 genes into the GEPIA website to obtain a box plot (Fig. 5A) of gene expression and concluded that the 7 genes are highly expressed in RCC. We also used the ChEA3 database to calculate the common transcription factors (Fig. 5B) of hub genes and found strong interaction between them. Finally, data quality control, standardization, data set dimensionality reduction, and cell classification were performed using the R software packages “seurat.” The TSNE and UMAP methods were used to visualize the cell types. A violin plot (Fig. 5C) was generated to observe the specific expression of hub genes in different cell clusters, the expression level of which are high in RCC and low in normal renal tissue. In normal kidney tissues, the hub gene was almost not expressed and SIRPG was not detected (Fig. 5B). Hub genes were mainly concentrated in clusters 0 and 5, which represented CD8 + T lymphocyte and T help cells, respectively. We mapped the gene expression to the UMAP results and visually assessed the specificity of gene expression (Fig. 5D). Finally, we studied the markers of the related genes to define the single-cell type of RCC (Fig. 5E). The hub genes were mainly expressed in CD8 + T lymphocytes, which confirmed our findings.

## 4. Discussion

Recently, an increasing number of studies have focused on TME since immunotherapies have shown high efficacy in cancer treatment.^[16]^ The ongoing in-depth research on the immunologic characteristics of the tumor microenvironment has facilitated the development of numerous innovative immunotherapeutic techniques, as well as the identification of potential clinically useful biomarkers. CD8 + T lymphocytes play an important role in the TME. Immune cells that target cancer seem to be CD8 + T lymphocytes.^[17]^ Based on CD8 + T lymphocyte-related genes, Dafeng Xu et al studied the prognostic characteristics of pancreatic cancer.^[18]^ Zhang et al established a new CD8 + T lymphocyte-related gene marker to assess the prognostic risk and immunotherapy response in patients with lung adenocarcinoma.^[19]^ Lalos et al found that high CD8 + T-cell density and SDF-1 expression represent distinct positive prognostic factors in colorectal cancer (CRC).^[20]^ However, few studies have constructed a CD8 + T lymphocyte-related gene prognostic model to identify marker genes related to CD8 + T lymphocytes in RCC.

WGCNA, an advanced single-cell analytical method, can be used to identify clusters (modules) of highly correlated genes, summarize such clusters using the module eigengene or an intramodular hub gene, relate modules to one another and external sample traits (via the eigengene network methodology), and calculate module membership measures. Correlation networks enable network-based gene-screening strategies to identify candidate biomarkers and therapeutic targets.^[21]^ Most studies have analyzed approximately 5000 genes; however, herein, we upgraded the hardware, innovatively included 10,000 genes for analysis, and improved the breadth and depth of gene selection.

CIBERSORT is an analytical technique developed by Newman et al to estimate the abundance of various cell types in a mixed-cell population using gene expression data. CIBERSORT is superior to other methods in terms of noise, unknown mixture content, and closely related cell types and has been commonly cited.^[22]^ We combined WGCNA and CIBERSORT, a popular bioinformatics algorithm, to screen hub genes.

In this study, we not only identified specific CD8 + T lymphocyte-related genes but also constructed a seven-gene prognostic model and verified our gene signature using single-cell RNA sequencing data,^[23]^ which set our study apart from previous studies. In the risk scoring model, a coefficient value > 0 (*SIRPG*, *PTPN7*, *APOBEC3G*, *FASLG* and *TIGIT*) indicated a negative correlation with prognosis, while < 0 value (*EOMES* and *CD3G*) indicated positive correlation.

We ranked the correlation coefficients of the 7 genes according to their absolute values to determine their roles in tumor prognosis. According to the findings of Leonard et al, *APOBEC3G* is a potential biomarker for tumor-infiltrating T cells with good outcomes for high-grade serous ovarian carcinoma. Although the *APOBEC3* enzymes play significant physiological functions in defending cells against endogenous and foreign DNA-based diseases, their dysregulation is linked to cancer.^[24]^ Based on 233 clinical samples Ting Peng et al confirmed the association between high *APOBEC3G* expression and poor prognosis in patients with RCC as well as a positive correlation between *APOBEC3G* and PD-L1 expression.^[25]^

The *CD3G* gene encodes the gamma chain of the T-cell receptor/CD3 complex, which is a crucial component in T-cell development and function. This complex plays a key role in the positive and negative selection of T cells, their effector functions, and the functions of regulatory T cells.^[26]^ *CD3G* deficiency leads to a decrease in the variety of regulatory T cells, an increase in their clonality, and a decrease in their ability to suppress immune responses. Patients with CD3G mutations exhibit a distinct genetic signature in their T-cell repertoire, which may be a contributing factor to the higher incidence of autoimmunity observed in these individuals.^[26]^ According to Wang et al, *CD3G* is a potential prognostic and immunotherapeutic biomarker in patients with cervical cancer.^[27]^ *CD3G* is a hub gene significantly associated with good prognosis in head and neck squamous cell carcinoma.^[28]^

The protein encoded by *EOMES*, a transcription factor essential for embryonic development of the mesoderm and central nervous system in vertebrates, may be required for the differentiation of effector CD8 + T cells involved in defense against viral infection.^[29]^ Dielmann et al found that higher *EOMES* mRNA expression was an independent prognostic factor for OS and PFS in patients with sorafenib.^[30]^ *EOMES* is a key transcription factor involved in regulating the development and function of CD4 T cells, promoting their differentiation into various effector cell types, such as Th1 and Th2.^[31]^

*PTPNs* are classified into 4 primary superfamilies and encoded by 103 genes.^[3]^ The Human Genome Organization Nomenclature Committee designated each *PTP* with an official gene name. The latter method designated 17 non-receptor *PTPs* from the largest family (class I) as *PTPN*.^[32]^ Differentially expressed *PTPN* family members are found in digestive system malignancies. *PTPN12* expression is increased in stomach adenocarcinoma and CRC, making it a potential diagnostic biomarker.^[33]^ The study discovered a correlation between *PTPN7* and the infiltration of immune cells, namely in “immuno-hot” tumors, which are distinguished by a substantial abundance of immune cells. These findings indicate that *PTPN7* likely has a function in regulating the immune response in the tumor microenvironment.^[34]^

*SIRPγ* is a protein that is unique to T-cells and is produced by the SIRPG gene. Genetic variations of this gene have been found to be associated with autoimmune disorders, specifically type 1 diabetes. T cells with low levels of *SIRPγ* exhibit increased pathogenicity in vivo, as demonstrated in a graft-versus-host disease model. This suggests that reduced expression of *SIRPγ* may contribute to the heightened activity of T cells in autoimmune disorders. *SIRPγ* is a crucial controller of T cell function in autoimmune disorders, and its levels of expression may impact the severity and advancement of the disease.^[35]^ Immunological landscape analysis demonstrated the heterogeneity of immune subtypes. For example, Wang et al established and validated genetic markers (*SIRPG*) for the immune microenvironment to predict the prognosis of patients with head and neck squamous cell carcinoma.^[28]^

*FASLG* belongs to the tumor necrosis factor superfamily. The main role of the encoded transmembrane protein is to initiate apoptosis through its interaction with FAS.^[36]^ The necroptosis-related genes (*FASLG* included) related signature serves as a unique prognostic predictor.^[37]^ Honghao established a 6-gene model, including *FASLG*, to predict breast cancer prognosis.^[38]^

*TIGIT* plays a crucial role in cancer immunology by mediating immune suppression. It is highly expressed on CD4 + T cells, CD8 + T cells, NK cells, and TILs, where it inhibits T cell activation and function through its ligand CD155, contributing to tumor immune evasion. Additionally, *TIGIT* is associated with NK cell exhaustion and T cell dysfunction, reducing their ability to attack tumor cells.^[39]^ The key prognostic factor *TIGIT* was verified to have a modest expression in healthy kidney tissues and was much higher expressed in RCC tissues. *TIGIT* expression levels are correlated with poor OS, PFS, and DSS in RCC.^[40]^

No studies have shown a strong association between these 7 genes and RCC. Herein, we found that 7 genes were selectively expressed in the CD8 + T lymphocytes of patients with RCC. In the ROC and DCA curves, the area under the curve was > 0.8. The predictive power of the nomogram was superior to that of the risk score model. Our analysis suggested that T, N, histologic grade, and sex were unrelated to prognosis. However, whether the interaction between these variables was related to prognosis remains to be determined, and we believe that additional independent risk factors should be identified in the future. In the single-cell analysis, we could not determine all cell types; therefore, all of them could not be labeled.

Future studies should conduct genetic testing on patients with RCC to determine the survival TME using this nomogram. In addition, we performed an enrichment analysis of hub genes and transcription factors, which also highlighted the direction for future research on prognostic markers of CD8 + T lymphocytes. In this study, lncRNA was filtered out during data cleaning, hence assessing the involvement of lncRNA in the prognosis of RCC is another research direction.

## 5. Conclusion

This study identified a seven-gene prognostic model associated with CD8 + T lymphocytes that may significantly influence risk stratification in patients with RCC. The model includes genes such as APOBEC3G, which is involved in RNA editing and immune response; CD3G, a key component of the T-cell receptor complex; EOMES, a transcription factor essential for CD8 + T cell function; PTPN7, which modulates T-cell activation; SIRPγ, involved in immune regulation; FASLG, which induces apoptosis in target cells; and TIGIT, an immune checkpoint receptor that inhibits T-cell activation. These genes not only serve as potential biomarkers for CD8 + T lymphocyte-related therapies but also provide insights into the complex interactions within the tumor microenvironment of RCC. Understanding their roles can enhance patient stratification and inform therapeutic strategies targeting the immune landscape of RCC.

## 6. Limitations

As our research was only based on the TCGA database, additional clinical investigations and biological tests remain warranted to validate our findings. Besides, cancer subtypes have not been further stratified.

Variations may arise from different subtypes of kidney cancer, but this study’s database does not classify kidney cancer into specific subtypes, making it impossible to further investigate results related to the subtypes of kidney cancer.

One limitation of this study is the availability of tumor staging data. Although we provide detailed T, N, and M classifications, the exact classification of certain patients may vary according to different institutional standards. Furthermore, due to the limited sample size, the statistical analysis of each classification may affect the generalizability of the results.

## Acknowledgments

We would like to sincerely express my gratitude to Professor Tao Jiang for his guidance. We acknowledge the support of the National Natural Science Foundation of China (82471655). We would like to thank Editage (www.editage.cn) for English language editing.

## Author contributions

**Conceptualization:** Jingbang Liu.

**Data curation:** Jingbang Liu.

**Formal analysis:** Jingbang Liu.

**Funding acquisition:** Tao Jiang.

**Investigation:** Jingbang Liu.

**Methodology:** Jingbang Liu.

**Project administration:** Jingbang Liu.

**Resources:** Jingbang Liu.

**Software:** Jingbang Liu.

**Supervision:** Jingbang Liu, Tao Jiang.

**Validation:** Jingbang Liu.

**Visualization:** Jingbang Liu.

**Writing – original draft:** Jingbang Liu.

**Writing – review & editing:** Jingbang Liu.

## Supplementary Material


